# Development and external validation of an interpretable machine learning-based model for obesity risk prediction in 2–18-year-old children and adolescents in Beijing and Tangshan

**DOI:** 10.7189/jogh.16.04031

**Published:** 2026-01-16

**Authors:** Mei Xue, Shufang Liu, Xiaoqian Zhang, Zhixin Zhang, Wenquan Niu

**Affiliations:** 1 Graduate School, Beijing University of Chinese Medicine, Beijing, China; 2 Department of Pediatrics, China-Japan Friendship Hospital, Beijing, China; 3 Department of Traditional Chinese Medicine, Beijing Children’s Hospital, Capital Medicine University, National Center for Children’s Health, Beijing, China; 4 Institute of Clinical Medical Sciences, China-Japan Friendship Hospital, Beijing, China; 5 Center for Evidence-Based Medicine, Capital Center for Children’s Health, Capital Medical University, Capital Institute of Pediatrics, Beijing, China

## Abstract

**Background:**

The multifactorial mechanisms driving childhood obesity, a global public health challenge, are yet to be fully elucidated. We aimed to develop and externally validate three widely applied machine learning models alongside logistic regression in 2–18-year-old children and adolescents in Beijing and Tangshan to predict obesity risk. As a further step, we wanted to interpret the optimised model and translate it into a web-based tool to inform clinical decision-making.

**Methods:**

We analysed data of 19 024 (training/testing) and 2410 (external validation) children and adolescents from Beijing and Tangshan, respectively. Using a set of factors including demographic, familial, socioeconomic, lifestyle, and perinatal variables, we developed four models (light gradient boosting machine, random forest, eXtreme gradient boosting (XGBoost), and logistic regression) and compared their predictive performance. After validation, we selected an optimised model and interpreted it using SHapley Additive exPlanations (SHAP) analysis. Then, we developed an online calculator with interpretable visualisations to enable real-time risk assessment.

**Results:**

The XGBoost model exhibited superior performance, with an area under the receiver operating characteristic curve (AUROC) of 0.875 on the external validation set, significantly outperforming the logistic regression model (AUROC = 0.718). To identify the minimal feature subset that maintained model efficacy, we incrementally incorporated predictors in the descending order of SHAP importance values while assessing key performance metrics (accuracy, AUROC, and F-beta score). This SHAP-based analysis identified nine key predictors of childhood obesity: birth length, paternal body mass index (BMI), maternal BMI, sleep duration, physical activity, birth weight, maternal age at delivery, delivery mode, and gestational age. The deployed online tool provides individualised risk probabilities and SHAP-derived explanations.

**Conclusions:**

The XGBoost model in our study was the superior ensemble learning method for predicting childhood obesity. The digital tool integrates this model and can help clinical practitioners determine individuals’ risk of childhood obesity.

The global prevalence of childhood obesity tripled between 1975 and 2016, making it a pressing public health crisis with far-reaching health, social, and economic implications [1]. By 2025, the global obesity prevalence among 5–19-year-olds reached 9.4% (188 million), surpassing the proportion of underweight children in this group (9.2%) [2]. In 2020, the prevalence of obesity in China was reported to be 3.6% among children <6 years and 7.9% among those aged 6–17 years [3]. This condition – with an aetiology involving interactions between heritable predisposition and modifiable environmental factors (*e.g.* excess gestational weight gain, parental obesity, and obesogenic lifestyle habits) – imposes a significant morbidity burden [4–6]. Frequently persisting into adulthood, childhood obesity leads to metabolic, cardiovascular, and psychosocial sequelae, ultimately resulting in a reduced life span of affected individuals [7–12].

Traditional analytical approaches, such as logistic regression, have been widely used to identify risk factors across a broad range of clinical endpoints, yet often fail to capture complex, nonlinear, or multifactor relationships, thereby constraining their predictive accuracy and clinical application [13,14]. More flexible extensions of traditional methods, such as generalised additive models that address single-dimensional nonlinear relationships, prove computationally cumbersome and difficult to interpret with high-dimensional datasets, especially when accounting for multi-factor interactions. By contrast, machine learning (ML) models have shown promise for enhancing disease prediction performance in handling these complex interactions. However, many existing studies lack external validation or clinical interpretability (the black box problem) and remain untranslated into actionable tools for frontline healthcare use [15–17].

To address these limitations, we developed and externally validated three widely applied ML models alongside logistic regression using a large-scale cohort and multidimensional predictors. Then, we employed SHapley Additive exPlanations (SHAP) to enhance model interpretability, identifying a minimal set of core predictors that retain high predictive efficacy. Lastly, we deployed a clinically actionable web-based tool that delivers individualised childhood obesity risk probabilities, while offering SHAP-derived explanations to inform clinical decision-making.

## METHODS

### Study design and participants

We conducted four cross-sectional surveys in 2019, 2020, 2022, and 2024 using stratified cluster sampling, targeting children and adolescents aged 2–18 years from Beijing and Tangshan, China. In the first survey (September to November 2019), we recruited 3–6-year-old children in Beijing by randomly selecting four (two urban, two suburban) out of 16 districts, with five kindergartens sampled per district. In the second survey (September to December 2020), we focused on preschool-aged children in Tangshan, with two of seven districts sampled and five kindergartens selected per district. The third survey (January 2022) was conducted in Pinggu district, Beijing, within eight primary and eighteen junior high schools. In the fourth survey (April to May 2024), again in Pinggu district, we enrolled children and adolescents aged 2–18 years from seven kindergartens, seven primary schools, and three junior high schools.

For analysis, we combined data from the 2019, 2022, and 2024 Beijing surveys to form the model development dataset (training/testing), while the 2020 Tangshan survey data served as an independent external validation set. Beijing and Tangshan differ in many aspects. For example, according to the 2022 census data, there are approximately 21.8 million people in Beijing and 7.7 million in Tangshan. There are 16 districts in Beijing and 7 in Tangshan, covering an area of 16 410 km^2^ and 13 472 km^2^, respectively. Per capita disposable income was RMB 77 400 in Beijing and RMB 39 600 in Tangshan.

We report our findings per the STROBE statement checklist (Table S1 in the **Online Supplementary Document**). The ethics committees of China-Japan Friendship Hospital (2018-93-K67, 2024-KY-084) and Beijing University of Chinese Medicine (2022BZYLL0906) provided ethical approval for this study. Written informed consent was obtained from the parents or guardians of study participants and the children and adolescents over the age of eight years.

### Data collection and variable definition

Trained health practitioners performed standardised anthropometric measurements (height in cm and weight in kg, both recorded to one decimal place) at baseline in participating kindergartens or schools. Weight and height were measured using a calibrated mechanical scale (Model RGZ-120; verification division e = 500g, Changzhou, China) and a calibrated SECA-213 stadiometer (SECA GMBH & Co. KG, Hamburg, Germany), respectively, across study sites. All practitioners and supervising teachers received standardised training to ensure consistency in procedures, measurement procedures, and methodological uniformity, and to minimise bias.

Besides body height and weight, additional data were collected using structured questionnaires covering five domains: demographics (age, sex, and date of birth); perinatal characteristics (gestational age, delivery mode, birth length, birth weight, infant feeding method, breastfeeding duration, and time to solid food addition); lifestyle factors (sleep duration, sedentary behavior, physical activity, consumption of sweet or fried foods, and midnight snack eating); allergy history (asthma, eczema, food/drug allergy); family background (household income, parental anthropometric measurements, parental education attainment, and parental reproductive age). We selected these factors based on existing literature and expert consensus to capture both known and potential determinants of childhood overweight or obesity [18–24].

To ensure the reliability and validity of our survey questionnaires, we ran a pilot test with 200 randomly selected participants before formal distribution, where the Cronbach’s alpha reliability coefficient exceeded 0.85.

### Obesity definition

We calculated the body mass index (BMI) as weight (kg) divided by height squared (m^2^). Weight status was determined using age- and sex-standardised BMI z-scores for children, calculated as the standard deviation (SD) difference between an individual’s BMI and the population mean. Based on World Health Organization (WHO) growth standards (2006 for children aged 0–5 years [25], 2007 reference for children aged 5–19 years [26]), we defined underweight as a BMI z-score < −2 SD, overweight as a BMI z-score > +1 SD, and obesity as a BMI z-score > +2 SD. Here, we excluded underweight and overweight children and adolescents, focusing exclusively on children with obesity or normal weight.

### Quality control

After extracting the data from the Wenjuanxing digital platform to which the data were uploaded by parents or legal guardians, we addressed incomplete responses or anomalous entries by prompting designated teachers and school health professionals to contact parents or guardians to either supplement missing information or verify questionable responses.

### Statistical analyses

#### Data processing

We preprocessed the data through seven steps, by: excluding features with >30% missing values; recoding outliers (defined as values beyond Q1 ± 1.5 × interquartile range (IQR), per the Tukey method) as missing values; applying multiple imputation by chained equations to fill missing data; utilising synthetic minority oversampling technique to mitigate class imbalance (~3.6:1 control-to-case ratio); removing multicollinear features (variance inflation factor >10); excluding of one variable from correlated pairs (Spearman’s *ρ* ≥ 0.9); selecting features related to childhood obesity using three methods (least absolute shrinkage and selection operator (LASSO), Boruta, and recursive feature elimination); random partitioning of the Beijing data set into training (70%) and testing (30%) cohorts.

#### Model selection

The three feature selection methods rely on different analytical approaches. The LASSO employs L1 regularisation to penalise less relevant features. Boruta uses a permutation-based approach to pinpoint statistically significant features. Recursive feature elimination iteratively removes the least informative features based on model importance scores, effectively reducing dimensionality. To enhance robustness and mitigate biases inherent in any single method, we adopted a consensus-based threshold – retaining only those features identified by at least two of the three methods (Figure S3 in the **Online Supplementary Document**). In practice, LASSO narrowed the feature set to 16 clinically relevant features, while Boruta and recursive feature elimination each identified all 25 candidate features.

#### Model evaluation

Besides logistic regression, we implemented three ensemble learning algorithms: light gradient boosting machine, random forest, and eXtreme gradient boosting (XGBoost). We selected these methods for their ability to handle high-dimensional epidemiological data, model complex nonlinear relationships, and feature importance estimation. All models were tuned *via* grid search with fivefold cross-validation (30 iterations). We assessed performance on the external testing set using the following metrics: accuracy, area under the receiver operating characteristic curve (AUROC), area under the precision-recall curve, Brier score, classification error, F-beta score, Matthews correlation coefficient, negative predictive value, positive predictive value, sensitivity, and specificity. Bootstrapped 1000 resamples were used to derive 95% confidence intervals (CIs) for all metrics. To avoid data leakage, feature selection and hyperparameter tuning (subsample, max depth, *etc*.) relied solely on training data, with no validation/testing information used.

To quantify the prediction model’s clinical utility, we performed a decision curve analysis that evaluated the model’s net benefit over a continuous range of threshold probabilities (0−100%) for both the testing set and external validation set. Then, we compared the net benefit against two default strategies: intervening in all participants or none. We visualised the relationship between the number of participants predicted to be high-risk and the actual number of observed positive cases across varying risk thresholds using a clinical impact curve.

#### Validation to improve readability

SHAP values were used to interpret the optimised model. Subgroup analyses were performed across three age groups (<6, 6 − 12, and >12) in years. Population-level feature importance and individualised prediction contributions were visualised using the two plots: waterfall plots decomposing per-feature risk attribution; directional force plots illustrating feature-specific risk modification. A parsimonious feature subset was derived by iteratively evaluating model performance using SHAP-ranked features.

A secure R Shiny-based web tool was developed for clinical deployment. User-input data generate real-time outputs: individualised obesity risk probability and a personalised SHAP waterfall plot visualising predictive contributors.

Continuous features are presented as mean (SD) or median (interquartile range or IQR), and categorical features as frequency (percentage). Between-group comparisons were performed using independent Student’s *t* tests, Mann-Whitney U tests, χ^2^ tests or Fisher exact tests, where appropriate. Statistical significance was defined as a two-tailed *P* < 0.05.

We conducted all analyses in *R*, version 4.3.3 (R Core Team, Vienna, Austria) *via* RStudio Desktop, version 2023.12.1, build 402 (Posit, Boston, Massachusetts, USA), and in Stata, version 18 (StataCorp LLC, TX, USA). Specifically, we used the ‘survey’, ‘mice’, ‘car’, ‘ggplot2’, ‘psych’, ‘caret’, ‘corrplot’, ‘DMwR’, ‘glmnet’, ‘Boruta’, ‘ggcor’, ‘mlr3’, ‘dplyr’, ‘precrec’, ‘praznik’, ‘mlr3verse’, ‘mlr3learners’, ‘mlr3extralearners’, ‘mlr3proba’, ‘mlr3tuning’, ‘mlr3filters’, ‘lightgbm’, ‘ranger’, ‘xgboost’, ‘pROC’, ‘kernelshap’, and ‘shapviz’ in *R*, and the ‘ttest’, ‘ranksum’, and ‘tabulate’ commands in Stata.

## RESULTS

**Figure 1:**
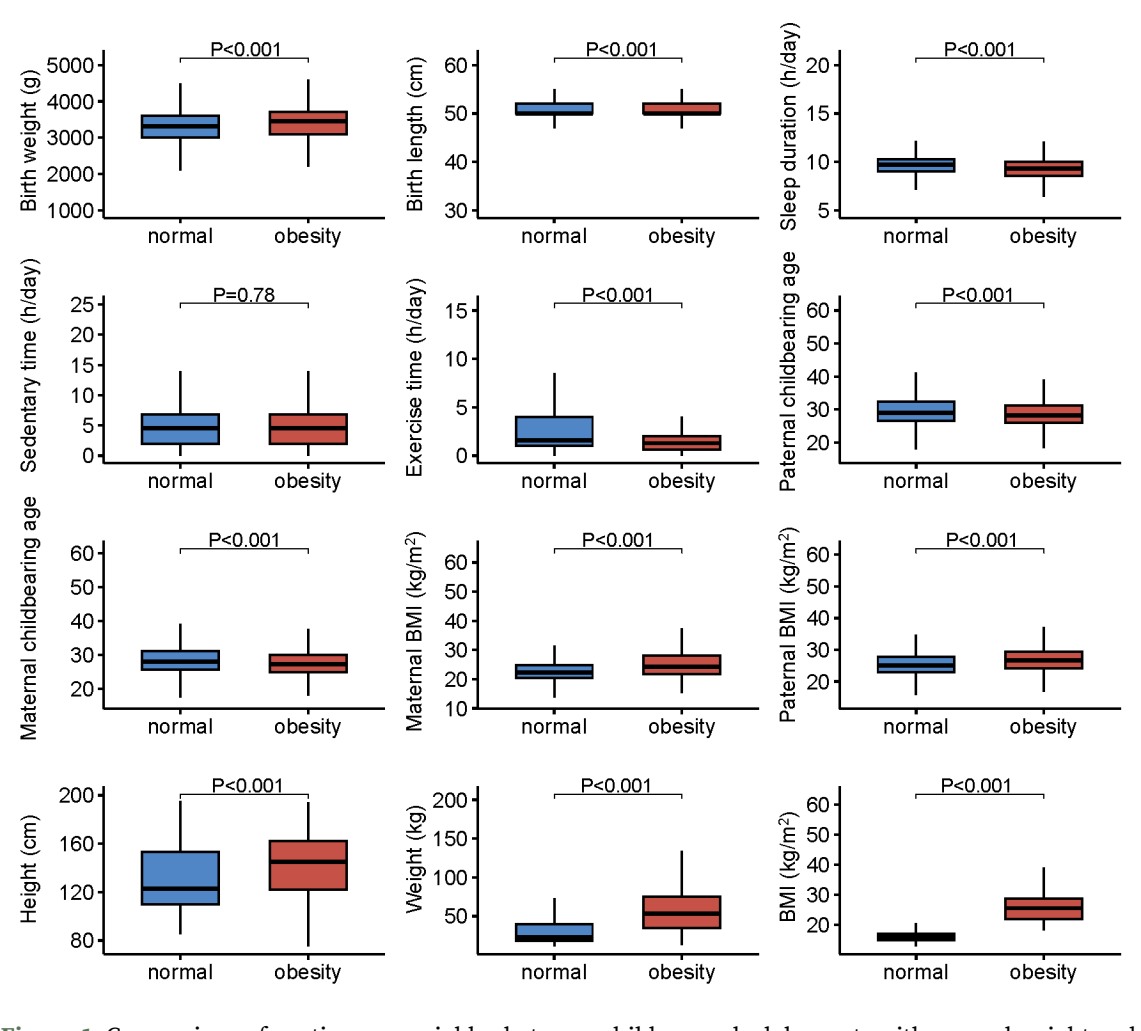
Comparison of continuous variables between children and adolescents with normal weight and obesity. BMI – body mass index. *Continuous data are expressed as mean (standard deviation) or median (interquartile range). *P*-values for the comparison between children and adolescents with normal weight and obesity were calculated by the independent Student’s *t*-test for normally distributed data and by rank-sum test for skewed data.

The analytical pipeline comprises three core panels: data processing, model selection, and model evaluation and application (Figure S1 in the **Online Supplementary Document**). We initially enrolled 24 912 children and adolescents aged 2–18 years, including those with normal weight, underweight, overweight, and obesity, of whom 4108 (16.5%) were classified as obese. After excluding underweight and overweight cases (Figure S2 in the **Online Supplementary Document**), we retained 16 614 participants from the Beijing development cohort and the 2410 from the Tangshan external validation cohort. Compared with children and adolescents with normal weight, those with obesity were, on average, older, had higher birthweight and parental BMI, shorter sleep duration, and less physical activity. A greater proportion of them were born *via* caesarean section, had mothers with lower educational attainment, and had a higher prevalence of asthma and eczema (**Figure 1**, **Table 1**).

**Table 1:** Comparison of categorical variables between children and adolescents with normal weight and obesity, n (%)

Characteristic	Normal weight (n = 14 916)	Obesity (n = 4108)	*P*-value*
**Age in years**			<0.001
2–6	6795 (45.55)	1044 (25.41)	
6–12	4586 (30.75)	1876 (45.67)	
12–18	3535 (23.70)	1188 (28.92)	
**Sex**			0.999
Boys	7356 (49.32)	2085 (50.75)	
Girls	7560 (50.68)	2023 (49.25)	
**Area**			<0.001
Beijing	12 836 (86.06)	3778 (91.97)	
Tangshan	2080 (13.94)	330 (8.03)	
**Delivery mode**			<0.001
Vaginal delivery	9562 (64.11)	1996 (48.59)	
Cesarean section	5354 (35.89)	2112 (51.41)	
**Gestational age in weeks**			0.003
<37	1338 (8.97)	440 (10.71)	
37–42	13 138 (88.08)	3545 (86.30)	
≥42	440 (2.95)	123 (2.99)	
**Infant feeding method**			0.006
Breast feeding	8819 (59.12)	2357 (57.38)	
Mixed feeding	4751 (31.85)	1316 (32.04)	
Artificial feeding	1346 (9.02)	435 (10.59)	
**Duration of breastfeeding in months**			<0.001
<6	3743 (25.09)	1218 (29.65)	
6–24	9359 (62.74)	2426 (59.06)	
≥24	1814 (12.16)	464 (11.30)	
**Time to add solid food**			<0.001
<6 months	2833 (18.99)	710 (17.28)	
6−9 months	10 664 (71.49)	2909 (70.81)	
≥9 months and later	1419 (9.51)	489 (11.90)	
**Maternal education**			<0.001
High school degree or below	5406 (36.24)	1764 (42.94)	
Bachelor’s degree	8279 (55.50)	2207 (53.72)	
Master’s degree or above	1231 (8.25)	137 (3.33)	
**Paternal education**			<0.001
High school degree or below	6258 (41.95)	2051 (49.93)	
Bachelor’s degree	7254 (48.63)	1904 (46.35)	
Master’s degree or above	1404 (9.41)	153 (3.72)	
**Family income (RMB per year)**			<0.001
<100000	5772 (38.70)	1853 (45.11)	
≥100000	9144 (61.30)	2255 (54.89)	
**Frequency of eating sweet food**			<0.001
Daily	1768 (11.85)	584 (14.22)	
Frequently (≥3 times/week)	5358 (35.92)	1706 (41.53)	
Occasionally (1–2 times/week)	5721 (38.35)	1322 (32.18)	
Seldom	2069 (13.87)	496 (12.07)	
**Frequency of eating fried food**			<0.001
Daily	2819 (18.90)	1002 (24.39)	
Frequently (≥ 3 times/week)	3203 (21.47)	1224 (29.80)	
Occasionally (1-2 times/week)	4550 (30.50)	986 (24.00)	
**Seldom**	4344 (29.12)	896 (21.81)	
**Eating midnight snacks**			<0.001
Yes	8476 (56.82)	2695 (65.60)	
No	6440 (43.18)	1413 (34.40)	
**Asthma**			<0.001
Yes	486 (3.26)	242 (5.89)	
No	14430 (96.74)	3866 (94.11)	
**Eczema**			0.620
Yes	3116 (20.89)	873 (21.25)	
No	11800 (79.11)	3235 (78.75)	
**Food/drug allergy**			0.980
Yes	3346 (22.43)	921 (22.42)	
No	11570 (77.57)	3187 (77.58)	

*χ^2^ test or Fisher’s exact test.

### Collinearity and correlation assessment

Regarding the correlation and collinearity analyses of continuous features (Figure S3 in the **Online Supplementary Document**), all variance inflation factor values were <5, indicating no substantial multicollinearity. Pairwise correlations among features were relatively weak (Spearman’s *ρ* < 0.9), and no features were excluded based on collinearity assessment.

### Model selection

We randomly partitioned the Beijing development dataset into training (70%) and testing (30%) subsets, while the Tangshan cohort served as the external validation set. For each ML model, we performed hyperparameter optimisation *via* grid search using fivefold cross-validation with 30 iterations – a strategy designed to select hyperparameters that balanced model complexity and predictive performance for subsequent validation (Table S2 in the **Online Supplementary Document**). Following hyperparameter tuning, we trained all ML models and the conventional logistic regression model on the training subset, with subsequent performance evaluated on both the testing and external validation sets.

The XGBoost model exhibited the best discriminative performance on the external validation set (**Table 2**), achieving the highest discriminative power (AUROC = 0.875), optimal accuracy (0.754), and the lowest Brier score (0.159), as well as a balanced sensitivity (0.645) and specificity (0.861). While the Tangshan cohort exhibited a slightly lower obesity prevalence (13.7%) compared with the Beijing cohort (22.7%), the XGBoost model maintained robust performance on the external validation set. While the sensitivity observed in the Tangshan cohort (0.645) was moderately lower than that in the internal testing set (0.818), the model achieved the highest discriminative ability (AUROC = 0.875) among all tested models.

**Table 2:** Performance comparison of three machine learning models and conventional logistic regression in predicting obesity on the testing and validation sets in children and adolescents*

Metric	LightGBM	Logistic regression	Random forest	XGBoost
Testing set				
*Accuracy*	0.779 (0.766–0.790)	0.697 (0.683–0.712)	0.785 (0.772–0.797)	0.771 (0.759–0.785)
*AUROC*	0.855 (0.844–0.866)	0.760 (0.746–0.774)	0.868 (0.857–0.878)	0.849 (0.838–0.861)
*AUPRC*	0.816 (0.797–0.835)	0.748 (0.729–0.768)	0.871 (0.858–0.883)	0.810 (0.791–0.829)
*Brier Score*	0.152 (0.146–0.157)	0.199 (0.194–0.205)	0.153 (0.148–0.158)	0.153 (0.147–0.159)
*Classification Error*	0.221 (0.210–0.234)	0.303 (0.288–0.317)	0.215 (0.203–0.228)	0.229 (0.215–0.241)
*F-beta*	0.788 (0.774–0.800)	0.699 (0.683–0.715)	0.789 (0.776–0.802)	0.782 (0.768–0.796)
*MCC*	0.559 (0.534–0.584)	0.394 (0.365–0.423)	0.570 (0.544–0.594)	0.545 (0.520–0.572)
*NPV*	0.823 (0.808–0.838)	0.703 (0.683–0.722)	0.806 (0.789–0.823)	0.818 (0.802–0.834)
*PPV*	0.756 (0.739–0.773)	0.696 (0.677–0.715)	0.773 (0.756–0.791)	0.749 (0.732–0.767)
*Sensitivity*	0.823 (0.808–0.838)	0.703 (0.683–0.722)	0.806 (0.789–0.823)	0.818 (0.802–0.834)
*Specificity*	0.823 (0.808–0.838)	0.703 (0.683–0.722)	0.806 (0.789–0.823)	0.818 (0.802–0.834)
Validation set				
*Accuracy*	0.738 (0.720–0.755)	0.650 (0.631–0.670)	0.668 (0.649–0.686)	0.754 (0.737–0.771)
*AUROC*	0.863 (0.847–0.877)	0.718 (0.696–0.739)	0.786 (0.767–0.804)	0.875 (0.860–0.889)
*AUPRC*	0.818 (0.792–0.843)	0.697 (0.666–0.727)	0.752 (0.722–0.782)	0.826 (0.799–0.852)
*Brier Score*	0.168 (0.160–0.177)	0.218 (0.211–0.225)	0.201 (0.194–0.208)	0.159 (0.151–0.167)
*Classification Error*	0.262 (0.245–0.280)	0.350 (0.330–0.369)	0.332 (0.314–0.351)	0.246 (0.229–0.263)
*F-beta*	0.701 (0.677–0.724)	0.603 (0.579–0.628)	0.596 (0.569–0.620)	0.723 (0.702–0.745)
*MCC*	0.490 (0.454–0.524)	0.307 (0.271–0.346)	0.357 (0.320–0.393)	0.519 (0.485–0.553)
*NPV*	0.618 (0.588–0.646)	0.536 (0.508–0.565)	0.492 (0.463–0.519)	0.645 (0.618–0.674)
*PPV*	0.810 (0.784–0.835)	0.691 (0.661–0.719)	0.755 (0.724–0.786)	0.821 (0.796–0.847)
*Sensitivity*	0.618 (0.588–0.646)	0.536 (0.508–0.565)	0.492 (0.463–0.519)	0.645 (0.618–0.674)
*Specificity*	0.857 (0.836–0.876)	0.763 (0.739–0.787)	0.843 (0.823–0.862)	0.861 (0.842–0.880)

AUROC – area under the receiver operating characteristic curve, AUPRC – area under the precision–recall curve, LightGBM – light gradient boosting machine, MCC – Matthews correlation coefficient, NPV – negative predictive value, PPV – positive predictive value, XGBoost – eXtreme gradient boosting

*Data are expressed as metric (95% confidence interval) derived from 1000 bootstrap resamples.

Bootstrap validation with 1000 resamples confirmed the consistent superiority of the XGBoost model, as seen from the distributions of receiver operating characteristic (ROC), AUROC, and area under the precision-recall curve with 95% CIs (Figure S4 in the **Online Supplementary Document**). The calibration curves for both the testing set and validation set showed excellent agreement between predicted probabilities and observed outcomes across the entire risk spectrum (Figure S5 in the **Online Supplementary Document**). The decision curve analysis plot showed that the prediction model yielded a superior net benefit in both the testing utility (Figure S6 in the **Online Supplementary Document**).

### Model interpretation

**Figure 2:**
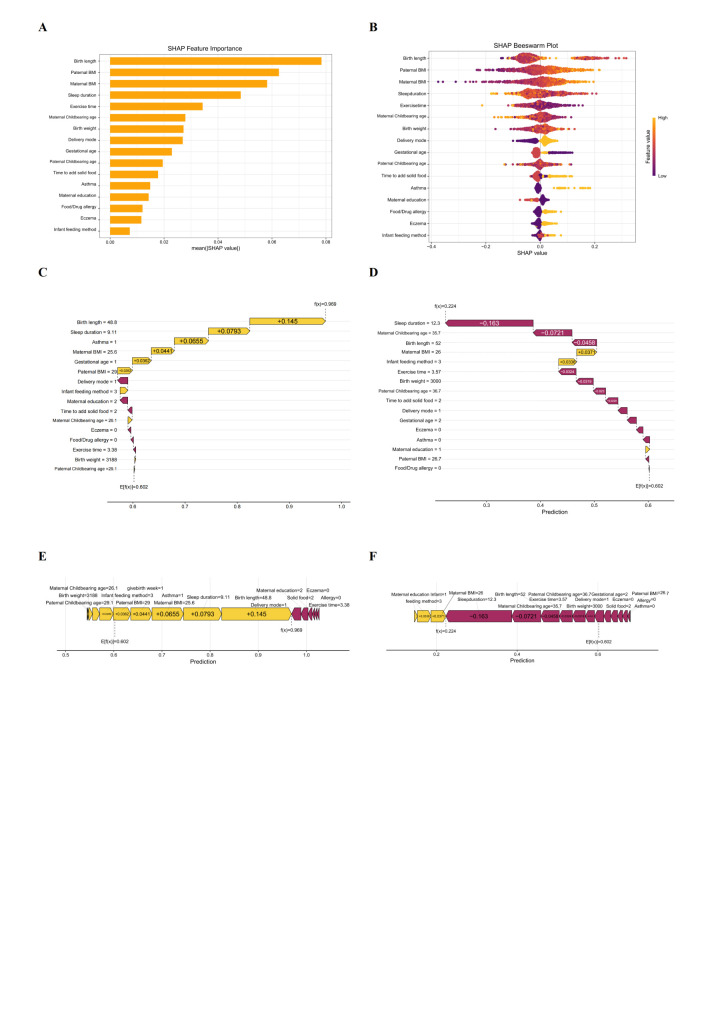
Global and local SHAP illustrations under the optimised XGBoost model. **Panel A.** SHAP importance plot of variables sorted by feature SHAP values in descending order. **Panel B. **SHAP bee swarm plot sorted by mean |SHAP| values (descending), with point colours indicating raw feature values (yellow = high, purple = low) and horizontal positions showing directional impact on obesity prediction (right = increases likelihood, left = decreases likelihood). **Panels C and D.** SHAP waterfall plot of features. **Panels E and F.** SHAP force plot of features. XGBoost – eXtreme Gradient Boosting, SHAP – SHapley Additive exPlanations.

Within the global interpretation of the XGBoost model (**Figure 2**, Panels A and B, birth length emerged as the most influential feature, followed in order by paternal BMI, maternal BMI, sleep duration, exercise time, maternal childbearing age, birth weight, delivery mode, gestational age, paternal childbearing age, timing of solid food introduction, asthma history, maternal education attainment, food/drug allergy history, eczema history, and infant feeding method.

We also established two case-specific interpretations *via* SHAP analyses that represent individual predictions on the validation cohort (**Figure 2**, Panels C and F). The first case involved a child with obesity (model output f(x) = 0.969 *vs*. the population mean of 0.602) with the following characteristics: birth length 48.8 cm, sleep duration 9.11 hours, positive asthma history, maternal BMI 25.6 kg/m^2^, preterm gestational age, paternal BMI 29 kg/m^2^, vaginal delivery, formula feeding, maternal education attainment at bachelor’s degree, solid food introduction at 6–9 months, maternal childbearing age 26.1 years, no eczema history, no food/drug allergies, daily exercise time 3.38 hours, birth weight 3188 g, and paternal childbearing age 29.1 years. Positive contributors to obesity risk included birth length, sleep duration, asthma status, maternal BMI, gestational age, paternal BMI, feeding method, maternal age at childbirth, birth weight, and paternal age at childbirth.

The second case involved a child with normal weight (f(x) = 0.224 *vs*. mean 0.602) with characteristics: sleep duration 12.3 hours, maternal childbearing age 35.7 years, paternal childbearing age 36.7 years, birth length 52 cm, birth weight 3000 g, maternal BMI 26 kg/m^2^, paternal BMI 26.7 kg/m^2^, mixed feeding method, daily exercise 3.57 hours, solid food introduction at 6–9 months, vaginal delivery, term gestation, no eczema history, no asthma history, maternal education at high school level or below, and no food/drug allergies. In this case, only maternal BMI, infant feeding method, and maternal education positively influenced obesity risk.

Among the optimal features selected by the optimised XGBoost model for obesity risk prediction, SHAP analyses revealed four common features in the top 10: paternal BMI, maternal BMI, exercise time, and birth weight. Notably, paternal BMI, maternal BMI, and exercise time consistently ranked within the top 5 across all age subgroups (Figure S7 in the **Online Supplementary Document**). Model performance plateaued after the top nine features, which were therefore retained for downstream analyses (**Table 3**). Figure S8 in the **Online Supplementary Document** displays the SHAP dependence plot for all features to facilitate understanding of how single features affected obesity prediction outcomes.

**Table 3:** Cumulative prediction performance of the optimised XGBoost model for obesity in children and adolescents

Top 16 features	Cumulative number of top 16 features	Accuracy	F-beta	AUROC
Birth length	1	0.672	0.752	0.698
Paternal BMI	2	0.709	0.740	0.781
Maternal BMI	3	0.733	0.753	0.800
Sleep duration	4	0.758	0.777	0.823
Exercise time	5	0.758	0.773	0.832
Maternal childbearing age	6	0.769	0.783	0.840
Birth weight	7	0.768	0.782	0.838
Delivery mode	8	0.767	0.781	0.842
Gestational age	9	0.774	0.787	0.843
Paternal childbearing age	10	0.770	0.782	0.845
Time to add solid food	11	0.771	0.783	0.848
Asthma	12	0.767	0.779	0.847
Maternal education	13	0.774	0.784	0.849
Food/drug allergy	14	0.771	0.782	0.849
Eczema	15	0.775	0.785	0.849
Infant feeding method	16	0.770	0.780	0.847

AUROC – area under the receiver operating characteristic curve, BMI – body mass index, XGBoost – eXtreme gradient boosting

### Model application

**Figure 3:**
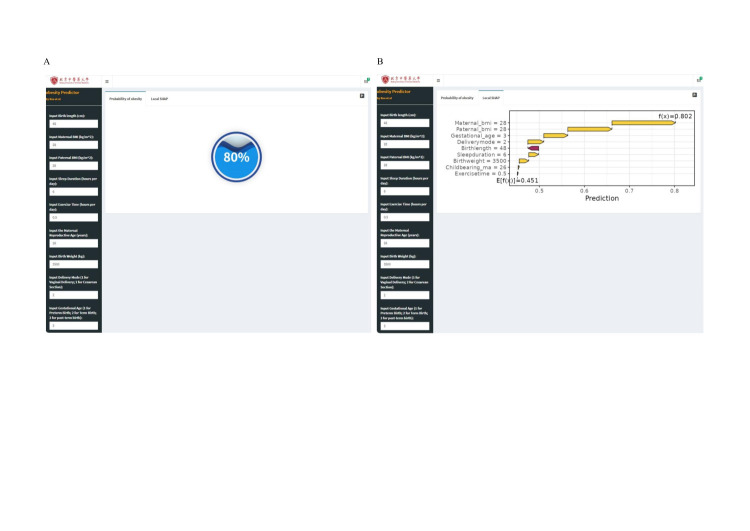
Deployment of an online prediction tool for obesity in children and adolescents under the optimised XGBoost model. **Panel A.** Estimated probabilities of obesity in children and adolescents under the optimised XGBoost model based on input features. **Panel B.** SHAP values of input features under the optimised XGBoost model based on input features. BMI – body mass index, SHAP – SHapley Additive exPlanations, XGBoost – eXtreme gradient boosting.

The online tool, based on the optimised XGBoost model and its key feature selection, offers two core functions: calculating individualised obesity risk probabilities and visualising feature importance *via* SHAP value analysis (**Figure 3**). Its interface comprises a left-hand input section for personal parameters and a right-hand output section that displays the risk assessment *via* a water balloon visualisation. The tool is now available for public use [27].

In a representative case that demonstrates the model’s utility to quantify obesity risk and deliver interpretable outputs, an individual with a birth length 48 cm, birth weight 3500 g, caesarean delivery, post-term gestation; maternal BMI 28 kg/m^2^, paternal BMI 28 kg/m^2^, maternal age at delivery 26 years, sleep duration of six hours per day, and physical activity 0.5 hours per day was estimated to have an 80% obesity risk. The model output (f(x) = 0.802) was substantially higher than the population mean of 0.451. Key positive contributors to the elevated risk included higher maternal and paternal BMI, post-term gestational age, caesarean delivery, shorter sleep duration, higher birth weight, and lower daily exercise time.

## DISCUSSION

We sought to develop an interpretable obesity prediction model for children and adolescents aged 2–18 years from Beijing and Tangshan, China, by comparing three ensemble ML models with conventional logistic regression. Using a large-scale native Beijing cohort with rigorous external validation in Tangshan, the optimised XGBoost model exhibited superior predictive performance. We further deployed the model within an interactive web application to facilitate its use in clinical contexts. The model’s innovation lies in two core aspects: it is grounded in region-specific Chinese paediatric data, ensuring alignment with local growth patterns, and its integrated SHAP analysis transforms it into an interpretable decision-support system. Taken together, this enables individualised risk factor decomposition to guide targeted interventions in clinical practice.

Obesity has become increasingly prevalent across all age groups, including children or adolescents and adults. Childhood obesity, for example, often persists into adulthood [28,29], making the elucidation of its underlying determinants a priority task that, despite existing research, still needs to be tackled. A major limitation in delineating robust risk profiles stems from statistical methodology – an area which is now being revolutionised by advances in artificial intelligence, which offers enhanced diagnostic precision and clinical decision-making capacity. Building on prior research using conventional analytical methods, our work surpasses traditional logistic regression approaches by implementing three robust ensemble ML models to better capture complex, nonlinear relationships among risk factors.

Our XGBoost model outperforms other models in external validation, achieving the highest AUROC (0.875) and competitive accuracy (0.754). The XGBoost has emerged as a cornerstone of clinical prediction modelling, including for obesity risk stratification, due to its robust regularisation to mitigate overfitting, scalable parallel computing efficiency, resilience to missing and sparse data, and inherent interpretability potentials [30–34].

To address the ‘black box’ challenge of ML, we integrated SHAP analysis, which quantifies global feature importance while enabling granular interpretation of individual predictions. This approach clarifies how specific feature combinations drive model outputs for each child or adolescent, bridging algorithmic complexity with clinically actionable insights. To translate these findings into practice, we developed an interactive web-based tool that computes individualised obesity risk probabilities in real time. User-input feature values generate instantaneous risk predictions alongside localised SHAP-driven visualisations, empowering clinicians and researchers with data-driven, interpretable decision support.

Our XGBoost model identified a hierarchical set of features for childhood obesity: birth length emerged as the most salient contributor, followed by parental BMI (paternal and maternal), lifestyles (sleep duration, exercise time), and perinatal determinants (birth weight, delivery mode, gestational age). The prominence of parental BMI as a high-level predictor strongly reinforces the well-established relationship between inherited predisposition and shared familial environments in obesity pathogenesis [35,36]. From a biological perspective, it reflects the transmission of obesity-susceptible genes (*e.g.* FTO, MC4R) that regulate energy homeostasis, appetite control, and adipocyte differentiation [37,38]. These genetic variants increase a child’s inherent adiposity risk and enhance sensitivity to obesogenic environments. Epidemiological evidence shows that children with one parent with obesity face a threefold higher adulthood obesity risk, escalating tenfold if both parents are obese [39].

In terms of lifestyle factors, inadequate sleep and reduced exercise time compound obesity risk by disrupting metabolic regulation and energy balance [19,40–43]. Sleep deprivation dysregulates hypothalamic-pituitary-adrenal axis function, altering leptin and ghrelin levels to increase appetite and energy intake [44,45]. Additionally, insufficient sleep exacerbates fatigue, reducing spontaneous physical activity and promoting sedentary behaviours. Perinatal variables (birth length, weight, gestational age, delivery mode, infant feeding method) may reflect foetal programming effects, where intrauterine growth patterns predispose to later adiposity [46,47]. For example, infants with longer birth lengths (often linked to intrauterine overgrowth) have reduced insulin sensitivity and blunted leptin responses in early childhood – adaptations favouring energy storage. This aligns with prior studies showing that large-for-gestational-age infants (often with longer birth lengths) are at an increased risk of obesity, distinct from small-for-gestational-age infants who develop obesity via catch-up growth [48,49]. Thus, birth length serves as a marker of intrauterine metabolic programming, shaping long-term obesity trajectories.

Maternal age at delivery and educational attainment may act as proxies for socioeconomic circumstances and caregiving environments, which in turn affect children’s dietary patterns and physical activity behaviours. To address this potential relation, we employed restricted cubic spline models within logistic regression to identify optimal threshold values. Existing evidence suggests that suboptimal reproductive age may exacerbate paediatric adiposity risk [50]. Our logistic regression identified a nonlinear association between parental age and offspring obesity risk, peaking at paternal ages of 24–30 years and maternal ages of 23–28 years, with subsequently diminishing risk at more advanced reproductive ages. Less prominent, but clinically relevant factors (asthma, eczema, allergy history) suggest immune-metabolic crosstalk, warranting further mechanistic exploration [20,21,51].

Our subgroup analyses revealed that parental BMI and exercise time maintained consistent predictive value across all age groups, underscoring their fundamental role in obesity risk assessment. This consistence highlights that inherited predisposition (reflected by parental BMI) and modifiable lifestyle factors (particularly physical activity) exert enduring influences throughout childhood development. The shared predictive significance of birth weight across models further reinforces the importance of early-life determinants in shaping long-term obesity trajectories. Collectively, these findings indicate a need for early-life interventions targeting modifiable determinants, while acknowledging immutable risks to stratify high-risk cohorts.

Lastly, the deployed web tool can be integrated easily into child healthcare workflows, such as to early risk identification during annual kindergarten/school entrance health examinations to guide school-family collaborative interventions, as well as in rapid risk stratification during routine community child health clinic visits, enabling referral of high-risk children to specialists. All features (*e.g.* birth parameters, parental BMI, lifestyle factors) we identified here are routinely collected during standard well-child visits or *via* simple questionnaires, meaning they require no additional or expensive tests and can be feasibly gathered in primary care settings. The tool’s usability is, therefore, further supported by its extremely low marginal cost and high potential for targeted prevention, but also by its SHAP-enabled interpretability, which can facilitate communication between healthcare providers and families by clarifying the ‘why’ behind the childhood obesity risk. Our future work will include usability testing and a prospective, cluster-randomised controlled trial to rigorously evaluate the tool’s real-world impact on clinical decision-making, patient behaviours, and childhood obesity incidence.

### Limitations

Several limitations should be considered when interpreting our findings. First, although BMI serves as a widely accepted metric for assessing nutritional status, as endorsed by the WHO, alternative anthropometric indices, such as waist-to-hip ratio, waist-to-height ratio, and the body roundness index, may more precisely estimate adiposity distribution. However, waist and hip circumference measurements were unavailable in our dataset. Second, childhood weight status is influenced by complex interactions of multiple features. The lack of biochemical or physiological markers prevented us from exploring any mediating pathways and underlying biochemical mechanisms. Third, our study population included only children and adolescents from Beijing and Tangshan, limiting the generalisability of our findings. Future work could explore calibration transfer techniques to adapt the model to new populations and semi-supervised learning to leverage abundant unlabelled data from new regions, reducing dependency on large fully annotated external datasets. Fourth, the cross-sectional design of our study precludes establishing causal relationships between risk features and childhood obesity. To mitigate this to an extent, we embedded penalised regression (*e.g.* LASSO regularisation) within ML models to enhance stability and reduce spurious correlations from correlated predictors. Fifth, excluding overweight and underweight children and adolescents enhanced the specificity of normal *vs*. obese comparisons, but limited the generalisability of our inferences to other groups. Future studies could include all weight status categories to explore continuous risk patterns.

## CONCLUSIONS

Our analysis confirms the XGBoost as a superior ensemble learning method for childhood obesity prediction. By identifying key influential features and integrating them into an accessible digital tool, this research bridges the gap between predictive modelling and practical healthcare applications. Our findings not only elucidate obesity risk profiles in Chinese paediatric populations, but also demonstrate the model’s potentials for early identification of high-risk children and adolescents in clinical settings.

## Additional material


Online Supplementary Document

## References

[1] Boutari C Mantzoros CSA 2022 update on the epidemiology of obesity and a call to action: as its twin COVID-19 pandemic appears to be receding, the obesity and dysmetabolism pandemic continues to rage on. Metabolism. 2022;133:155217. 10.1016/j.metabol.2022.155217 35584732 PMC9107388

[2] Iacobucci G Childhood obesity more common than underweight for first time in history, Unicef reports. BMJ. 2025;390:r1915. 10.1136/bmj.r1915 40935575

[3] Pan XF Wang L Pan A Epidemiology and determinants of obesity in China. Lancet Diabetes Endocrinol. 2021;9:373–92. 10.1016/S2213-8587(21)00045-0 34022156

[4] Raab R Michel S Günther J Hoffmann J Stecher L Hauner H Associations between lifestyle interventions during pregnancy and childhood weight and growth: a systematic review and meta-analysis. Int J Behav Nutr Phys Act. 2021;18:8. 10.1186/s12966-020-01075-7 33413486 PMC7792105

[5] González-Domínguez Á Jurado-Sumariva L Domínguez-Riscart J Saez-Benito A González-Domínguez R Parental obesity predisposes to exacerbated metabolic and inflammatory disturbances in childhood obesity within the framework of an altered profile of trace elements. Nutr Diabetes. 2024;14:2. 10.1038/s41387-024-00258-6 38238301 PMC10796909

[6] Pan X Jiang C Wang W Lin J Lifestyle factors associated with being overweight and obesity in children and adolescents: a cross-sectional study in Zhejiang, China. Front Public Health. 2025;13:1551099. 10.3389/fpubh.2025.1551099 40177075 PMC11961437

[7] Abbasi A Juszczyk D van Jaarsveld CHM Gulliford MC Body Mass Index and Incident Type 1 and Type 2 Diabetes in Children and Young Adults: A Retrospective Cohort Study. J Endocr Soc. 2017;1:524–37. 10.1210/js.2017-00044 29264507 PMC5686575

[8] Park H Choi JE Jun S Lee H Kim HS Lee HA Metabolic complications of obesity in children and adolescents. Clin Exp Pediatr. 2024;67:347–55. 10.3345/cep.2023.00892 37986568 PMC11222907

[9] Pinhas-Hamiel O Hamiel U Bendor CD Bardugo A Twig G Cukierman-Yaffe T The Global Spread of Severe Obesity in Toddlers, Children, and Adolescents: A Systematic Review and Meta-Analysis. Obes Facts. 2022;15:118–34. 10.1159/000521913 35016185 PMC9021657

[10] Shaunak M Byrne CD Davis N Afolabi P Faust SN Davies JH Non-alcoholic fatty liver disease and childhood obesity. Arch Dis Child. 2021;106:3–8. 10.1136/archdischild-2019-318063 32409495

[11] Lindberg L Danielsson P Persson M Marcus C Hagman E Association of childhood obesity with risk of early all-cause and cause-specific mortality: A Swedish prospective cohort study. PLoS Med. 2020;17:e1003078. 10.1371/journal.pmed.1003078 32187177 PMC7080224

[12] Marcus C Danielsson P Hagman E Pediatric obesity-Long-term consequences and effect of weight loss. J Intern Med. 2022;292:870–91. 10.1111/joim.13547 35883220 PMC9805112

[13] Chen Z Dazard JE Salerno P Sirasapalli SK Makhlouf MH Rajagopalan S Composite socio-environmental risk score for cardiovascular assessment: An explainable machine learning approach. Am J Prev Cardiol. 2025;22:100964. 10.1016/j.ajpc.2025.100964 40200918 PMC11976227

[14] Wu LH Zhao D Niu JY Fan QL Peng A Luo CG Development and validation of multi-center serum creatinine-based models for noninvasive prediction of kidney fibrosis in chronic kidney disease. Ren Fail. 2025;47:2489715. 10.1080/0886022X.2025.2489715 40230189 PMC12001852

[15] Lim H Lee H Kim J A prediction model for childhood obesity risk using the machine learning method: a panel study on Korean children. Sci Rep. 2023;13:10122. 10.1038/s41598-023-37171-4 37344518 PMC10284805

[16] Liu H Leng Y Wu YC Chau PH Chung TWH Fong DYT Robust identification key predictors of short- and long-term weight status in children and adolescents by machine learning. Front Public Health. 2024;12:1414046. 10.3389/fpubh.2024.1414046 39381765 PMC11458556

[17] Wang Y Shi S Wei X Wu Y Shi Y Cai J Application and Analysis of Random Forest and Support Vector Classification in Risk Prediction of Childhood Obesity and Hyperuricemia. Diabetes Metab Syndr Obes. 2025;18:2221–33. 10.2147/DMSO.S519284 40655165 PMC12254580

[18] Chen K Zheng F Zhang X Wang Q Zhang Z Niu W Factors associated with underweight, overweight, and obesity in Chinese children aged 3-14 years using ensemble learning algorithms. J Glob Health. 2025;15:04013. 10.7189/jogh.15.04013 39913538 PMC11804908

[19] Gråstén A Tremblay MS Ortega FB Jaakkola T Yli-Piipari S Alnuaimi J Physical activity strategies for preventing school-aged children’s overweight and obesity: a systematic review. J Public Health Policy. 2025;46:484–502. 10.1057/s41271-025-00571-z 40442501

[20] Kuniyoshi Y Association between allergic diseases and body composition in toddlers: A Nationwide birth cohort study in Japan. Pediatr Allergy Immunol. 2025;36:e70163. 10.1111/pai.70163 40762152

[21] Umano GR Pistone C Tondina E Moiraghi A Lauretta D Miraglia Del Giudice E Pediatric Obesity and the Immune System. Front Pediatr. 2019;7:487. 10.3389/fped.2019.00487 31824900 PMC6883912

[22] Zhang J Zhai Y Feng XQ Li WR Lyu YB Astell-Burt T Gender Differences in the Prevalence of Overweight and Obesity, Associated Behaviors, and Weight-related Perceptions in a National Survey of Primary School Children in China. Biomed Environ Sci. 2018;31:1–11. 29409580 10.3967/bes2018.001

[23] Zhang X Wang Q Gao Z Zhang Z Wu J Zhang Z Prevalence of malnutrition and its associated factors among 18,503 Chinese children aged 3-14 years. Front Nutr. 2023;10:1228799. 10.3389/fnut.2023.1228799 38148792 PMC10750408

[24] Zhang Y Wang Q Xue M Pang B Yang M Zhang Z Identifying factors associated with central obesity in school students using artificial intelligence techniques. Front Pediatr. 2022;10:1060270. 10.3389/fped.2022.1060270 36533227 PMC9748186

[25] World Health Organization. Body mass index-for-age (BMI-for-age). 2026. Available: https://www.who.int/toolkits/child-growth-standards/standards/body-mass-index-for-age-bmi-for-age. Accessed: 5 January 2026.

[26] World Health Organization. BMI-for-age (5–19 years). 2026. Available: https://www.who.int/tools/growth-reference-data-for-5to19-years/indicators/bmi-for-age. Accessed: 5 January 2026.

[27] Xue M Lui S Zhang X Zhang Z Niu WObesity Predictor. Available: https://xueapplication.shinyapps.io/obesityrisk/. Accessed: 9 January 2026.

[28] Simmonds M Llewellyn A Owen CG Woolacott N Predicting adult obesity from childhood obesity: a systematic review and meta-analysis. Obes Rev. 2016;17:95–107. 10.1111/obr.12334 26696565

[29] The Lancet Diabetes Endocrinology Childhood obesity: a growing pandemic. Lancet Diabetes Endocrinol. 2022;10:1. 10.1016/S2213-8587(21)00314-4 34863372 PMC9765420

[30] Montomoli J Romeo L Moccia S Bernardini M Migliorelli L Berardini D Machine learning using the extreme gradient boosting (XGBoost) algorithm predicts 5-day delta of SOFA score at ICU admission in COVID-19 patients. J Intensive Med. 2021;1:110–6. 10.1016/j.jointm.2021.09.002 36785563 PMC8531027

[31] Moore A Bell M XGBoost, A Novel Explainable AI Technique, in the Prediction of Myocardial Infarction: A UK Biobank Cohort Study. Clin Med Insights Cardiol. 2022;16:11795468221133611. 10.1177/11795468221133611 36386405 PMC9647306

[32] Schindele A Krebold A Heiß U Nimptsch K Pfaehler E Berr C Interpretable machine learning for thyroid cancer recurrence predicton: Leveraging XGBoost and SHAP analysis. Eur J Radiol. 2025;186:112049. 10.1016/j.ejrad.2025.112049 40096773

[33] Wiens M Verone-Boyle A Henscheid N Podichetty JT Burton J A Tutorial and Use Case Example of the eXtreme Gradient Boosting (XGBoost) Artificial Intelligence Algorithm for Drug Development Applications. Clin Transl Sci. 2025;18:e70172. 10.1111/cts.70172 40067353 PMC11895769

[34] Görmez Y Yagin FH Yagin B Aygun Y Boke H Badicu G Prediction of obesity levels based on physical activity and eating habits with a machine learning model integrated with explainable artificial intelligence. Front Physiol. 2025;16:1549306. 10.3389/fphys.2025.1549306 40740428 PMC12308079

[35] Carter S Parsons C Ward K Clynes M Dennison EM Cooper C Body mass index, prudent diet score and social class across three generations: evidence from the Hertfordshire Intergenerational Study. BMJ Nutr Prev Health. 2021;4:36–41. 10.1136/bmjnph-2020-000178 34308110 PMC8258032

[36] Cooper R Hyppönen E Berry D Power C Associations between parental and offspring adiposity up to midlife: the contribution of adult lifestyle factors in the 1958 British Birth Cohort Study. Am J Clin Nutr. 2010;92:946–53. 10.3945/ajcn.2010.29477 20702606

[37] Noor N Cardenas A Rifas-Shiman SL Pan H Dreyfuss JM Oken E Association of Periconception Paternal Body Mass Index With Persistent Changes in DNA Methylation of Offspring in Childhood. JAMA Netw Open. 2019;2:e1916777. 10.1001/jamanetworkopen.2019.16777 31880793 PMC6991200

[38] Sivakumar S Lama D Rabhi N Childhood obesity from the genes to the epigenome. Front Endocrinol (Lausanne). 2024;15:1393250. 10.3389/fendo.2024.1393250 39045266 PMC11263020

[39] Andriani H Liao CY Kuo HW Parental weight changes as key predictors of child weight changes. BMC Public Health. 2015;15:645. 10.1186/s12889-015-2005-x 26164227 PMC4499442

[40] Alkhatib A Effects of Nutrition and Physical Activity Lifestyle Interventions on Childhood Obesity. Nutrients. 2025;17:2100. 10.3390/nu17132100 40647205 PMC12252048

[41] Men J Wang P Gao Q Li Y Zhu G Yu Z Impact of exercise on anthropometric outcomes in children and adolescents with overweight or obesity: a systematic review and meta-analysis based on 113 randomized controlled trials worldwide. BMC Public Health. 2025;25:2400. 10.1186/s12889-025-23413-9 40624670 PMC12232561

[42] St-Onge MP The role of sleep duration in the regulation of energy balance: effects on energy intakes and expenditure. J Clin Sleep Med. 2013;9:73–80. 10.5664/jcsm.2348 23319909 PMC3525993

[43] Tubbs AS Khader W Fernandez F Grandner MA The common denominators of sleep, obesity, and psychopathology. Curr Opin Psychol. 2020;34:84–8. 10.1016/j.copsyc.2019.11.003 31835070 PMC9190766

[44] Akhlaghi M Kohanmoo A Sleep deprivation in development of obesity, effects on appetite regulation, energy metabolism, and dietary choices. Nutr Res Rev. 2025;38:4–24. 10.1017/S0954422423000264 37905402

[45] Figorilli M Velluzzi F Redolfi S Obesity and sleep disorders: A bidirectional relationship. Nutr Metab Cardiovasc Dis. 2025;35:104014. 10.1016/j.numecd.2025.104014 40180826

[46] Gishti O Gaillard R Manniesing R Abrahamse-Berkeveld M van der Beek EM Heppe DH Fetal and infant growth patterns associated with total and abdominal fat distribution in school-age children. J Clin Endocrinol Metab. 2014;99:2557–66. 10.1210/jc.2013-4345 24712569

[47] Mook-Kanamori DO Durmuş B Sovio U Hofman A Raat H Steegers EA Fetal and infant growth and the risk of obesity during early childhood: the Generation R Study. Eur J Endocrinol. 2011;165:623–30. 10.1530/EJE-11-0067 21775498

[48] Dong Y Luo ZC Nuyt AM Audibert F Wei SQ Abenhaim HA Large-for-Gestational-Age May Be Associated With Lower Fetal Insulin Sensitivity and β-Cell Function Linked to Leptin. J Clin Endocrinol Metab. 2018;103:3837–44. 10.1210/jc.2018-00917 30032199 PMC6179169

[49] Gaskins RB LaGasse LL Liu J Shankaran S Lester BM Bada HS Small for gestational age and higher birth weight predict childhood obesity in preterm infants. Am J Perinatol. 2010;27:721–30. 10.1055/s-0030-1253555 20408111 PMC2949419

[50] Deng R Lou K Zhou SL Li XX Zou ZY Ma YH [Relationship between parental reproductive age and the risk of overweight and obesity in offspring]. Zhonghua Yu Fang Yi Xue Za Zhi. 2022;56:583–9. 35644971 10.3760/cma.j.cn112150-20220223-00171

[51] Calcaterra V Verduci E Ghezzi M Cena H Pascuzzi MC Regalbuto C Pediatric Obesity-Related Asthma: The Role of Nutrition and Nutrients in Prevention and Treatment. Nutrients. 2021;13:3708. 10.3390/nu13113708 34835964 PMC8620690

