# Supplementary material for: Development and external validation of an interpretable machine learning-based model for obesity risk prediction in 2–18-year-old children and adolescents in Beijing and Tangshan
Source: J Glob Health. 2026 Jan 16;16:04031. doi: 10.7189/jogh.16.04031 (PMC12810588; doi:10.7189/jogh.16.04031)
Supplement: Online Supplementary Document [file jogh-16-04031-s001.pdf]

Supplement to: Xue M, Liu S, Zhang X, Zhang Z, Niu W. Development and external validation of an interpretable machine learning-based model for obesity risk prediction in 2–18-year-old children and adolescents in Beijing and Tangshan. J Glob Health. 2026;16:04031.

Table S1. STROBE Statement—Checklist of items that should be included in reports of cross-sectional studies.

Table S2. Optimal hyperparameters of three machine learning models assessed in this study.

Figure S1. Selection flowchart of children and adolescents in this study.

Figure S2. Feature selection strategies by using the Spearman's correlation, VIF, LASSO, Boruta, and RFE methods.

Figure S3. The analytic pipeline of this machine learning-based study.

Figure S4. Performance comparison of three machine learning models versus logistic regression for childhood obesity on the external validation set.

Figure S5. Calibration curves of the optimized XGBoost model in predicting obesity on the testing and validation sets in children and adolescents.

Figure S6. Decision curve analysis (A) and clinical impact curve (B−C) of the optimized XGBoost model on the testing and validation sets.

Figure S7. Global SHAP analysis of the optimized XGBoost model across different age groups

Figure S8. Dependence of the SHAP-value-based features under the optimized XGBoost model on the validation set.

Table S1. STROBE Statement—Checklist of items that should be included in reports of ***cross-sectional studies***

Section	Item No	Recommendation	Page No
**Title and abstract**	1	(*a*) Indicate the study's design with a commonly used term in the title or the abstract	1
		(*b*) Provide in the abstract an informative and balanced summary of what was done and what was found	2
**Introduction**			
Background/rationale	2	Explain the scientific background and rationale for the investigation being reported	2−3
Objectives	3	State specific objectives, including any prespecified hypotheses	3
**Methods**			
Study design	4	Present key elements of study design early in the paper	3−4
Setting	5	Describe the setting, locations, and relevant dates, including periods of recruitment, exposure, follow-up, and data collection	3−4
Participants	6	(*a*) Give the eligibility criteria, and the sources and methods of selection of participants	3−4
Variables	7	Clearly define all outcomes, exposures, predictors, potential confounders, and effect modifiers. Give diagnostic criteria, if applicable	4−5
Data sources/ measurement	8*	For each variable of interest, give sources of data and details of methods of assessment (measurement). Describe comparability of assessment methods if there is more than one group	4−5
Bias	9	Describe any efforts to address potential sources of bias	5
Study size	10	Explain how the study size was arrived at	3−4
Quantitative variables	11	Explain how quantitative variables were handled in the analyses. If applicable, describe which groupings were chosen and why	5−6
Statistical methods	12	(*a*) Describe all statistical methods, including those used to control for confounding	5−7
		(*b*) Describe any methods used to examine subgroups and interactions	7
		(*c*) Explain how missing data were addressed	5−6
		(*d*) If applicable, describe analytical methods taking account of sampling strategy	3−7
		(*e*) Describe any sensitivity analyses	6−7
**Results**			
Participants	13*	(a) Report numbers of individuals at each stage of study—eg numbers potentially eligible, examined for eligibility, confirmed eligible, included in the study, completing follow-up, and analysed	8
		(b) Give reasons for non-participation at each stage	3−4
		(c) Consider use of a flow diagram	Figure S1
Descriptive data	14*	(a) Give characteristics of study participants (eg demographic, clinical, social) and information on exposures and potential confounders	8

		(b) Indicate number of participants with missing data for each variable of interest	NA
Outcome data	15*	Report numbers of outcome events or summary measures	8−10
Main results	16	(*a*) Give unadjusted estimates and, if applicable, confounder-adjusted estimates and their precision (eg, 95% confidence interval). Make clear which confounders were adjusted for and why they were included	7−9
		(*b*) Report category boundaries when continuous variables were categorized	8
		(*c*) If relevant, consider translating estimates of relative risk into absolute risk for a meaningful time period	NA
Other analyses	17	Report other analyses done—eg analyses of subgroups and interactions, and sensitivity analyses	10
**Discussion**			
Key results	18	Summarise key results with reference to study objectives	11
Limitations	19	Discuss limitations of the study, taking into account sources of potential bias or imprecision. Discuss both direction and magnitude of any potential bias	14
Interpretation	20	Give a cautious overall interpretation of results considering objectives, limitations, multiplicity of analyses, results from similar studies, and other relevant evidence	13−14
Generalisability	21	Discuss the generalisability (external validity) of the study results	13−14
**Other information**			
Funding	22	Give the source of funding and the role of the funders for the present study and, if applicable, for the original study on which the present article is based	15

*Give information separately for exposed and unexposed groups.

Note: An Explanation and Elaboration article discusses each checklist item and gives methodological background and published examples of transparent reporting. The STROBE checklist is best used in conjunction with this article (freely available on the Web sites of PLoS Medicine at the website http://www.plosmedicine.org/, Annals of Internal Medicine at http://www.annals.org/, and Epidemiology at the website http://www.epidem.com/). Information on the STROBE Initiative is available at the website www.strobe-statement.org.

Table S2. Optimal hyperparameters of three machine learning models assessed in this study.

Model	Ranges and optimal settings of inherited hyperparameters
LightGBM	
learning rate	[0.01, 1] and 0.12
bagging fraction	[0.1, 1] and 0.6
max depth	[1, 10] and 7
num leaves	[10, 1000] and 560
min data in leaf	[1, 100] and 12
feature fraction	[0.5, 1] and 0.83
Random forest	
ntree	[50, 500] and 350
mtry	[1, 10] and 7
nodesize	[1, 10] and 1
XGBoost	
nrounds	[50, 200] and 200
eta	[0.01, 0.2] and 0.14
max depth	[1, 10] and 5
min child weight	[1, 5] and 5
subsample	[0.1, 1] and 0.93
colsample bytree	[0.1, 1] and 0.7

Abbreviations: LightGBM, light gradient boosting machine; XGBoost, eXtreme gradient boosting.

Figure S1. Selection flowchart of children and adolescents in this study.

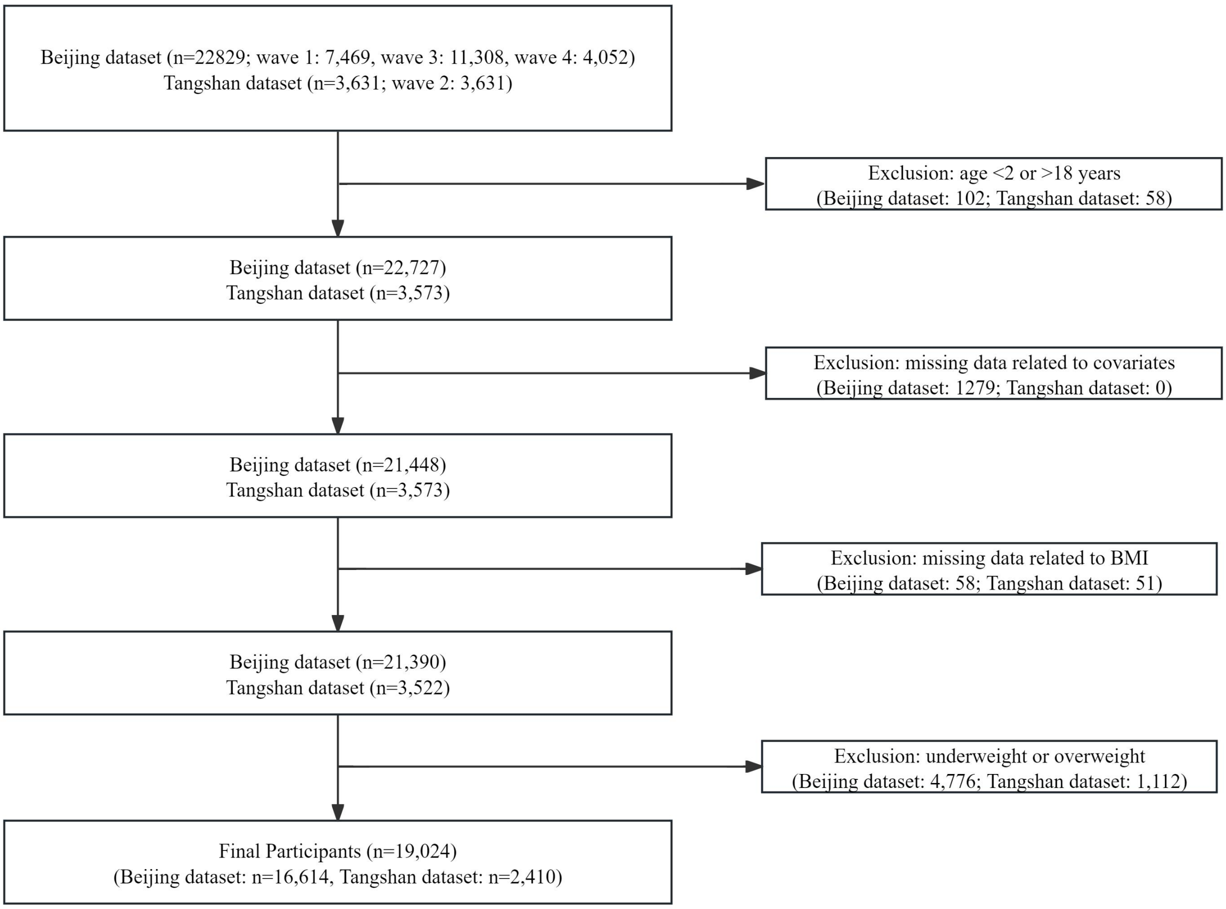

Figure S2. Feature selection strategies by using the Spearman's correlation, VIF, LASSO, Boruta, and RFE methods.

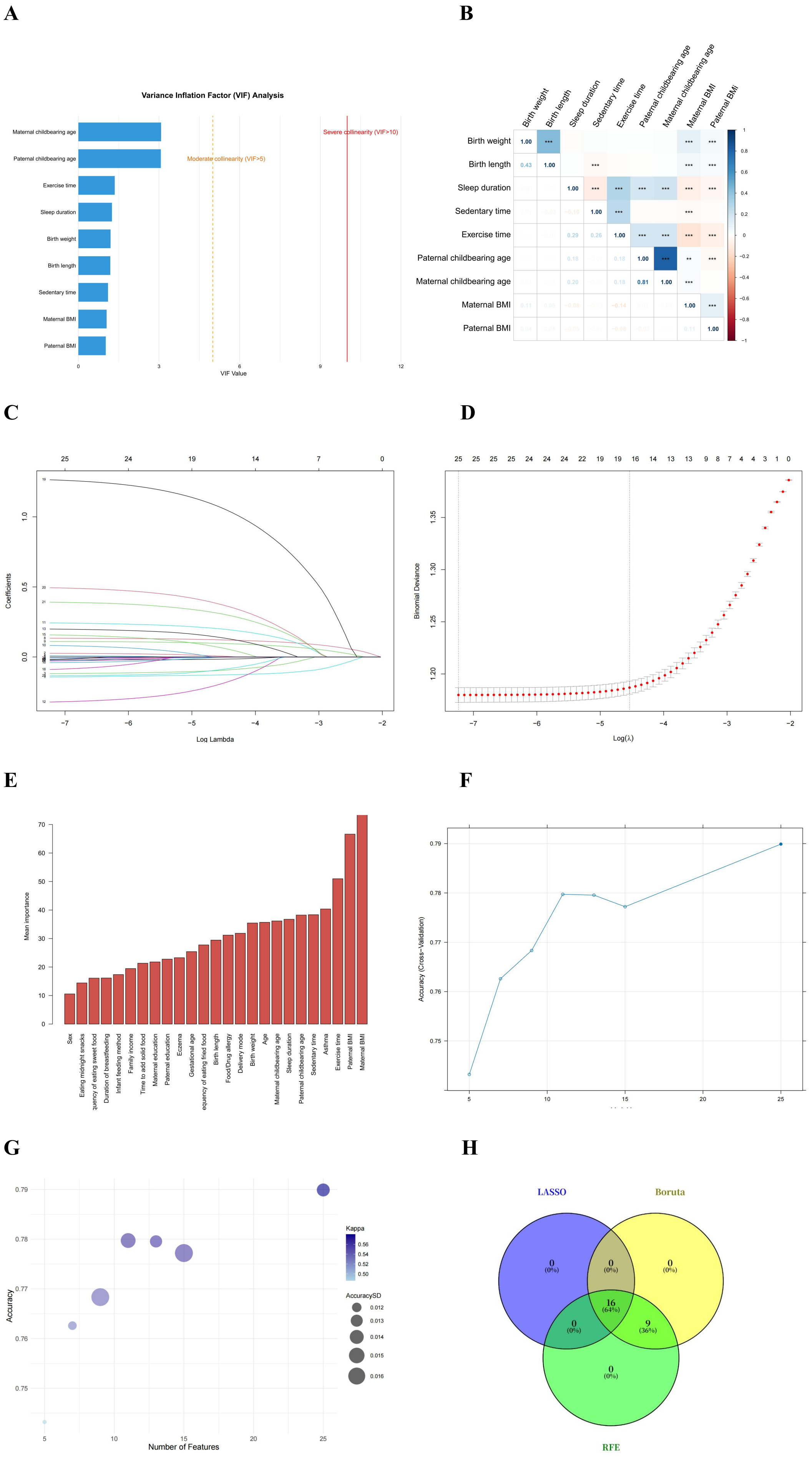

Abbreviations: VIF, variance inflation factor; LASSO, least absolute shrinkage and selection operator; RFE, recursive feature elimination.

Panel A: VIF distribution. Categorizing Collinearity Levels by VIF Thresholds of 5 and 10. VIF >10 indicates severe multicollinearity. Panel B: Spearman's correlation heat map. Dark blue shades indicate stronger positive correlations, and dark red shades represent stronger negative correlations. If correlation coefficient between two features is greater than 0.9, they are considered to have very strong correlation. Panel C: LASSO regression with L1 regularization was applied for feature selection, progressively sparsifying model coefficients. The colored trajectories depict the evolution of individual coefficient estimates across regularization intensities. Increasing the penalty term (log lambda) drives most coefficients toward zero, with only the most predictive features retaining non-zero values. Panel D: Binomial deviance vs. log(x) relationship analysis with red scatter points representing raw data observations and the gray smoothing curve capturing the trend of deviance variation across the dynamic range of x. Panel E: Stability of importance during model iterations. Panel F: Feature ranking via Boruta algorithm. Panel F: Cross-validation accuracy improves with the increase in the number of features. Panel G: Optimal number of features identified by RFE. Panel H: Venn diagram comparing feature selection by LASSO, Boruta, and RFE.

Figure S3. The analytic pipeline of this machine learning-based study.

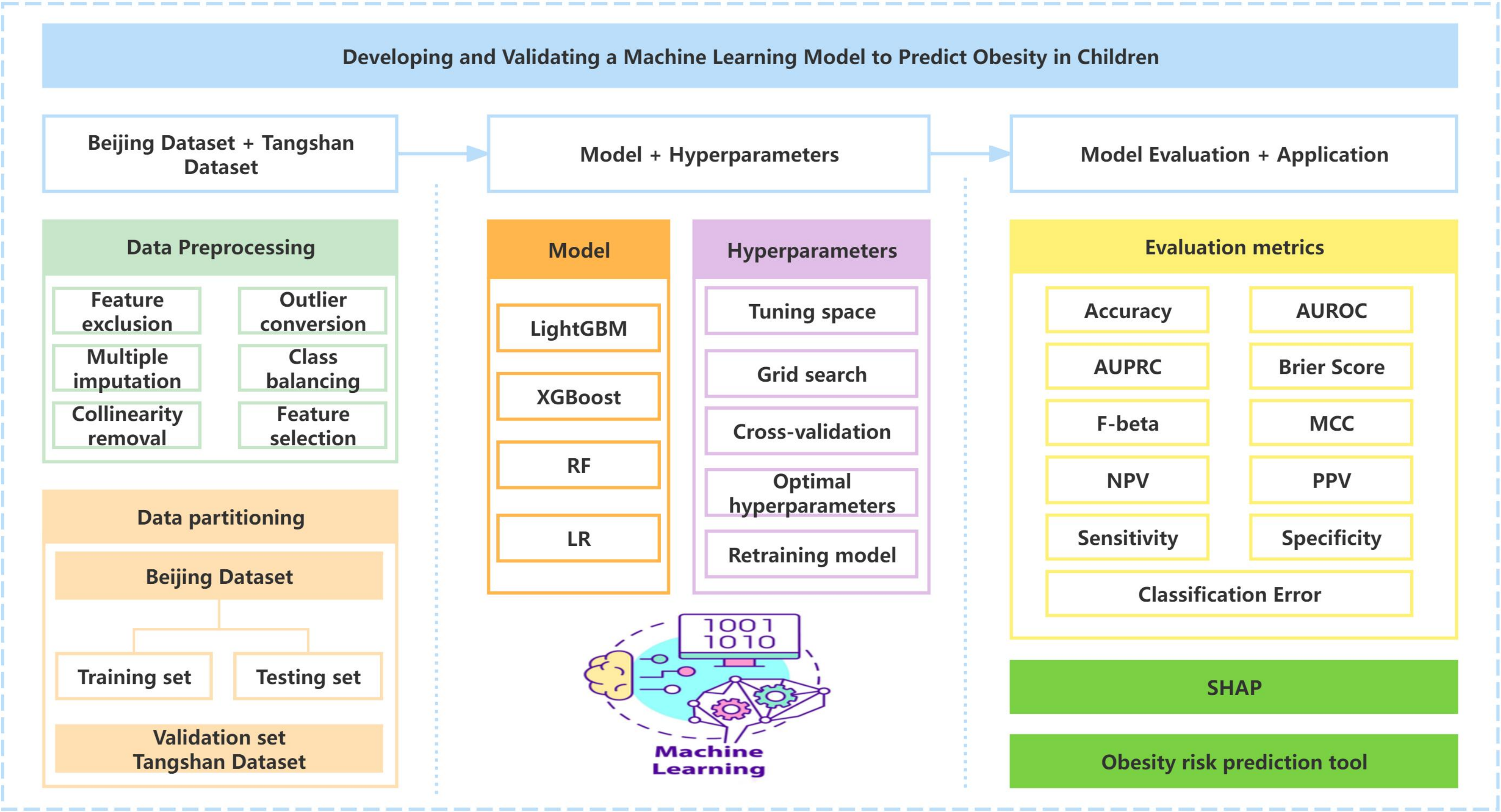

Abbreviations: AUROC, area under the receiver operating characteristic curve; AUPRC, area under the precision-recall curve; MCC, Matthes correlation coefficient; NPV, negative predictive value; PPV, positive predictive value; SHAP, SHapley Additive exPlanations.

Figure S4. Performance comparison of three machine learning models versus logistic regression for childhood obesity on the external validation set.

A **B** **C**

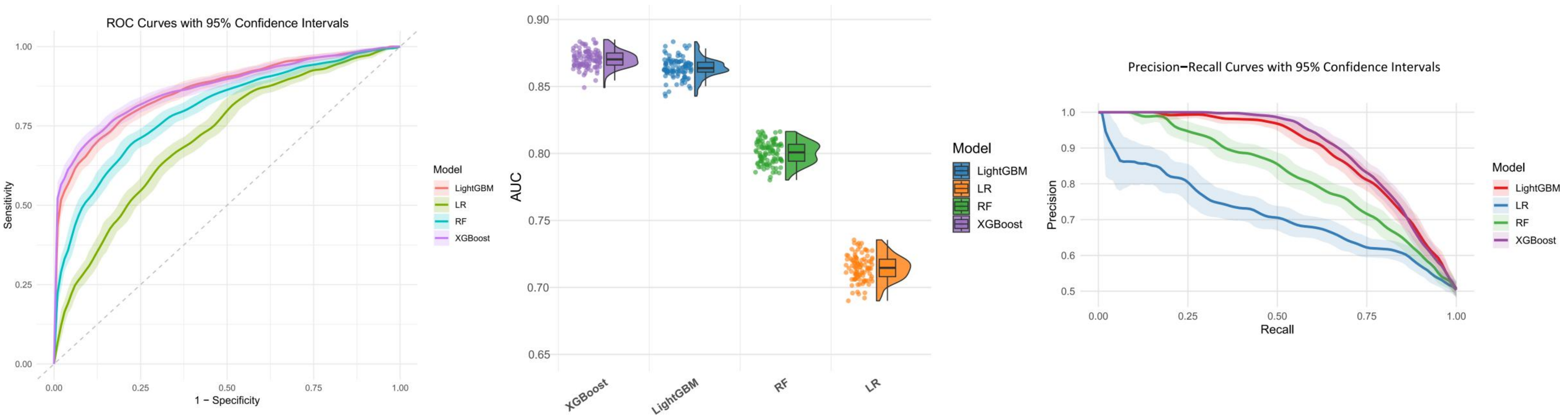

Abbreviations: AUC, area under the receiver operating characteristic (ROC) curve; AUPRC, area under the precision-recall curve (AUPRC); LightGBM, light gradient boosting machine; LR: logistic regression; PRC, precision-recall curve; RF, random forest; XGBoost, eXtreme gradient boosting.

Panels A: ROC curves with AUC values on the validation set.

Panels B: Raincloud plots combining boxplots (center line: median, box: interquartile range), kernel density distributions, and individual data points representing cross-validation folds.

Panels C: PRC with AUPRC values on the validation set.

Figure S5. Calibration curves of the optimized XGBoost model in predicting obesity on the testing and validation sets in children and adolescents.

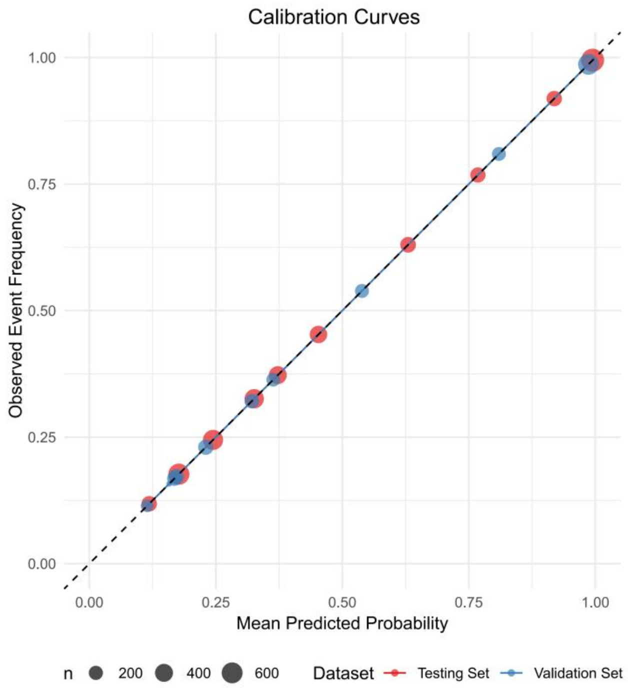

Solid lines represent the observed calibration curves for the testing set (red) and external validation set (blue), while dashed diagonal line indicates the ideal calibration reference.

Figure S6. Decision curve analysis (A) and clinical impact curve (B−C) of the optimized XGBoost model on the testing and validation sets.

A

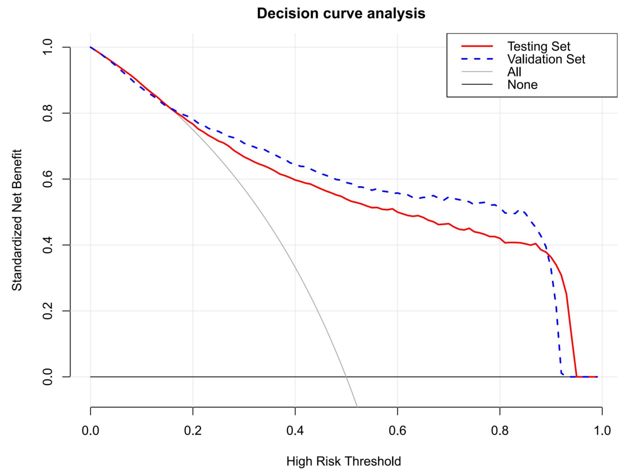

B

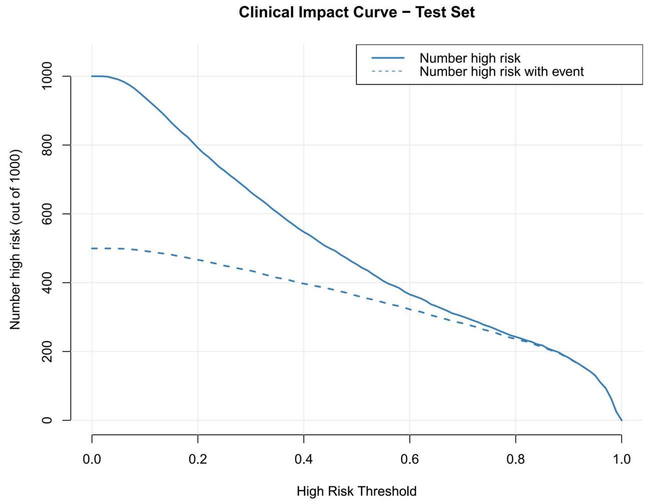

C

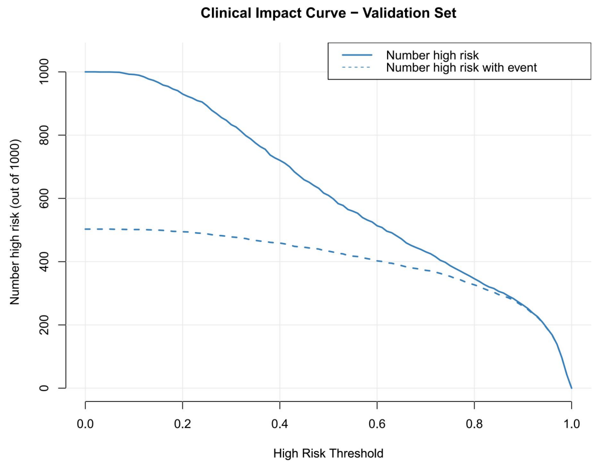

Abbreviations: XGBoost, eXtreme gradient boosting.

Figure S7. Global SHAP analysis of the optimized XGBoost model across different age groups

A

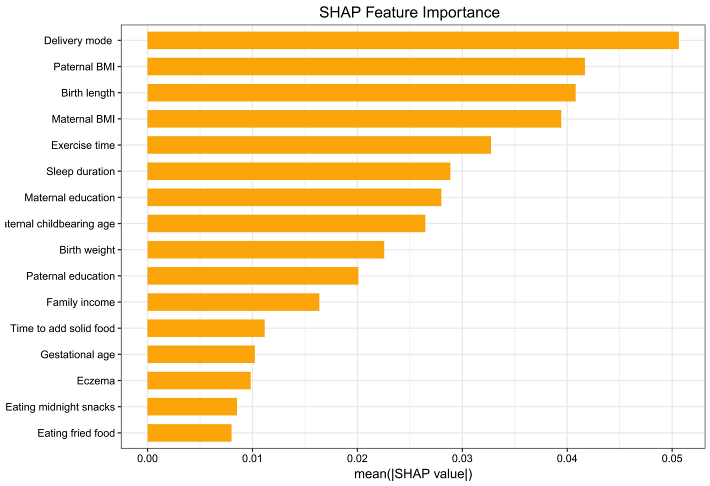

B

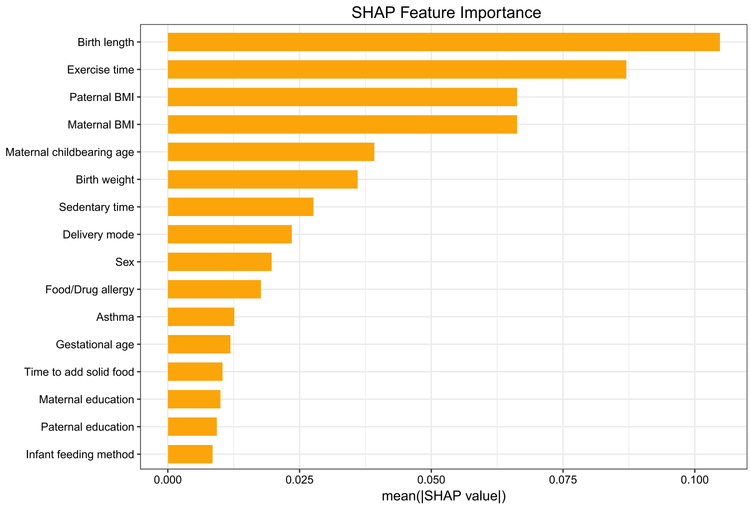

C

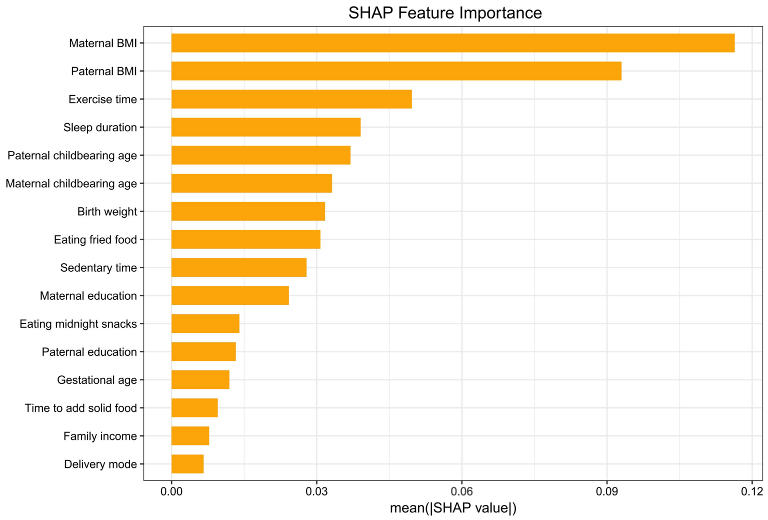

Abbreviations: SHAP, SHapley Additive exPlanations; XGBoost, eXtreme gradient boosting.

Panel A: SHAP importance plot of variables sorted by feature SHAP values in children under 6 years old.

Panel B: SHAP importance plot of variables sorted by feature SHAP values in children aged 6−12 years old.

Panel C: SHAP importance plot of variables sorted by feature SHAP values in children and adolescents aged ≥12 years old.

Figure S8. Dependence of the SHAP-value-based features under the optimized XGBoost model on the validation set.

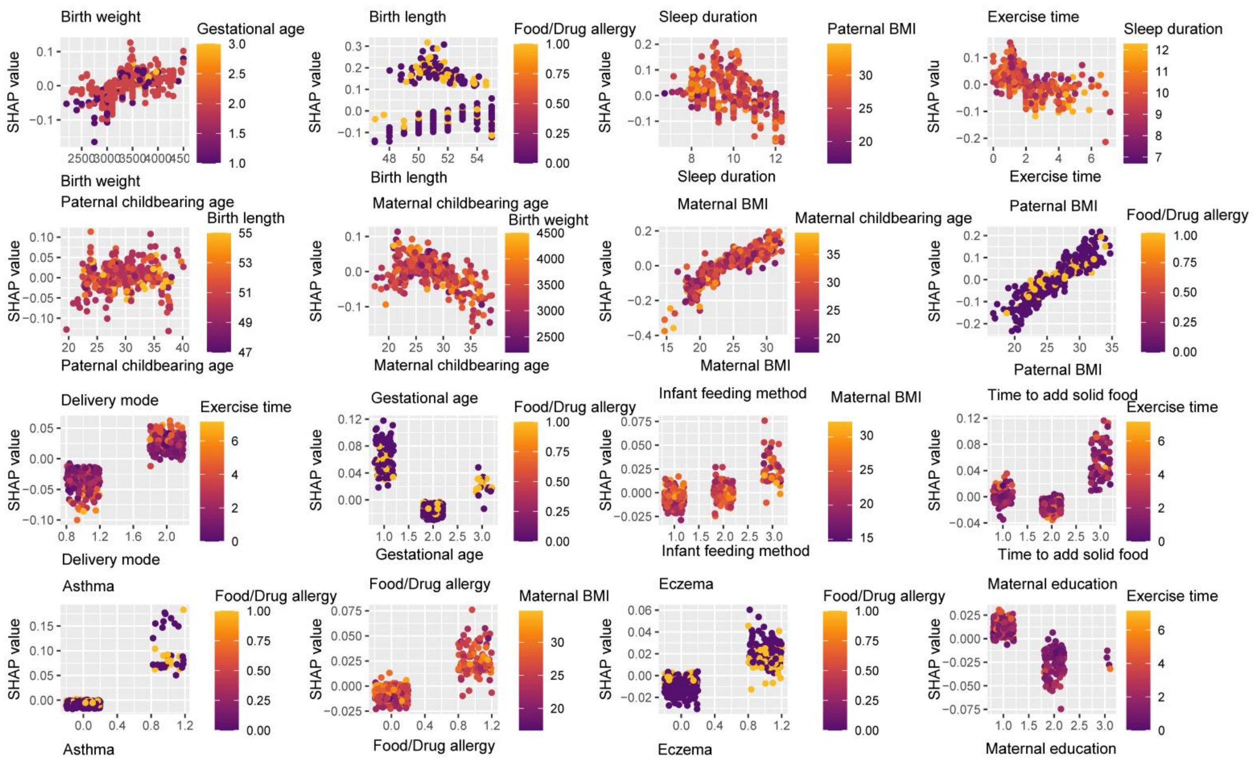

Abbreviations: BMI, body mass index; SHAP, SHapley Additive exPlanations; XGBoost, eXtreme gradient boosting.
